# Schiff Base-Crosslinked Tetra-PEG-BSA Hydrogel: Design, Properties, and Multifunctional Functions

**DOI:** 10.3390/jfb16020069

**Published:** 2025-02-18

**Authors:** Yuanyuan Qu, Jinlong Li, Xin Jia, Lijun Yin

**Affiliations:** 1 College of Food Science and Nutritional Engineering, China Agricultural University, 17 Qinghua Donglu, Haidian District, Beijing 100083, China; qu@cau.edu.cn (Y.Q.); xinjia@cau.edu.cn (X.J.); 2 College of Chemistry and Materials Engineering, Beijing Technology and Business University, 11 Fucheng Road, Haidian District, Beijing 100048, China

**Keywords:** hydrogel, Schiff base, BSA, Tetra-PEG, protein

## Abstract

Hydrogel network structures play a crucial role in determining mechanical properties and have broad applications in biomedical and industrial fields. Therefore, their rational design is essential. Herein, we developed a Schiff base-crosslinked hydrogel through the reaction of Tetra-armed polyethylene glycol with aldehyde end groups (Tetra-PEG-CHO) and bovine serum albumin (BSA) under alkaline conditions. In addition, the Tetra-PEG-BSA hydrogel showed a rapid gelation time of around 11 s, much faster than that of the GLU-BSA, HT-BSA, and GDL-BSA hydrogels. It had high optical transmittance (92.92% at 600 nm) and swelling ratios superior to the other gels in different solutions, maintaining structural integrity even in denaturing environments such as guanidine hydrochloride and SDS. Mechanical tests showed superior strain at break (84.12 ± 0.76%), rupture stress (28.64 ± 1.21 kPa), and energy dissipation ability (468.0 ± 34.9 kJ·m^−3^), surpassing all control group hydrogels. MTT cytotoxicity assays indicated that cell viability remained >80% at lower concentrations, confirming excellent biocompatibility. These findings suggest that Tetra-PEG-BSA hydrogels may serve as effective materials for drug delivery, tissue engineering, and 3D printing.

## 1. Introduction

Hydrogels are three-dimensional polymeric materials that can swell, but not dissolve in aqueous media, allowing them to absorb high amounts of water without losing their mechanical features [1,2]. Recently, protein-derived hydrogels that can be naturally sourced have gained great attention, especially naturally sourced hydrogel-based proteins, because of their biocompatibility, biodegradability, and wide range of functionalities [3,4,5]. Various functions, such as high strength, super-absorbability, self-healing/self-recovery ability, fatigue resistance, and even stimuli-responsiveness for discoloration, have been developed. Compared with conventional synthetic polymer hydrogels, protein-based hydrogels offer improved biocompatibility and bioavailability, making them ideal candidates for tissue engineering, drug delivery, tissue repair, wearable electronics, and various (bio)sensing applications [6,7,8,9].

However, as the architecture of native proteins is not very complex, their hydrated gel typically shows relatively weak mechanical responses compared to synthetic-based hydrogels, which hampers their usage as multifunctional biomaterials [10]. To address this limitation, researchers have employed various strategies to enhance the mechanical performance of protein hydrogels. Recent advancements in these strategies include physicochemical hybrid crosslinking, double crosslinking, double-network (DN) hydrogels, nanocomposites (NCs), and also topological networks, which can significantly improve hydrogels’ elasticity, strength, and toughness [11,12,13,14].

Bovine serum albumin (BSA) is a great candidate to form hydrogels with good properties owing to its natural biocompatibility, biodegradability, and functional groups for crosslinking, which can provide a lot of reactive sites to construct stable and tunable network structures [15,16]. To improve the mechanical properties of BSA-based hydrogels, an approach has been developed for chemical or enzymatic crosslinking and blending with synthetic polymers; however, simultaneously achieving high strength and toughness along with complete degradation and compatibility remains a challenge. The prevailing challenge of BSA hydrogel optimization is to identify effective and non-toxic crosslinkers [2]. For example, glutaraldehyde is widely used, but it has some toxicity concerns. Hence, the focus has shifted towards finding less toxic crosslinkers, which could also be biocompatible, for safer biomedical usage [8,12].

Among various non-toxic crosslinking strategies, PEG-based systems are especially appealing due to their enhanced biocompatibility, present FDA approval, and metabolic elimination from the body [17,18]. PEG hydrogels, however, often lack sufficient mechanical strength. To circumvent this limitation, PEG–protein hybrid gels have been designed, which would inherit the advantages of both (PEG′s role as a toxic and biocompatible modifier, with proteins like BSA presenting biological functionality, fulfilling molecular recognition) [2,19]. For instance, such networks can be formed when tetra-armed PEG (Tetra-PEG) containing aldehyde functional groups is crosslinked with amino functional groups of BSA to promote rapid gelation kinetics, better swelling behavior, high optical transmittance, and mechanical properties such as toughness and anti-fatigue features [20].

**Figure 1:**
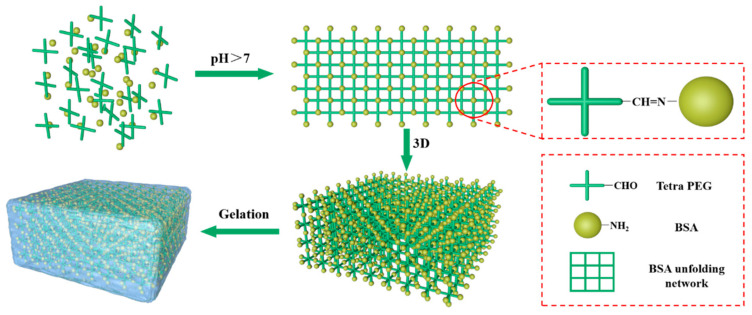
Mechanistic diagram of BSA and Tetra-PEG synthesis.

In this study, we detailed the characterization of a new BSA-Tetra-PEG hydrogel (Figure 1) and compared it to three different control groups: BSA–glutaraldehyde gels, heat-induced gels, and GDL-induced gels. The detailed characterizations of gel formation time, light transmittance, swelling properties in various solutions, mechanical properties, and microstructures will help to optimize Tetra-PEG-BSA hydrogels for a broad range of applications. Bio-friendly and wide-ranging PEG-BSA hybrid-type hydrogels can be manufactured, leading to enticing contenders for food, medicine, and biomedical life sciences, especially for enhanced tissue engineering and regenerative medicine.

## 2. Results and Discussion

### 2.1. Grafting Degree

**Figure 2:**
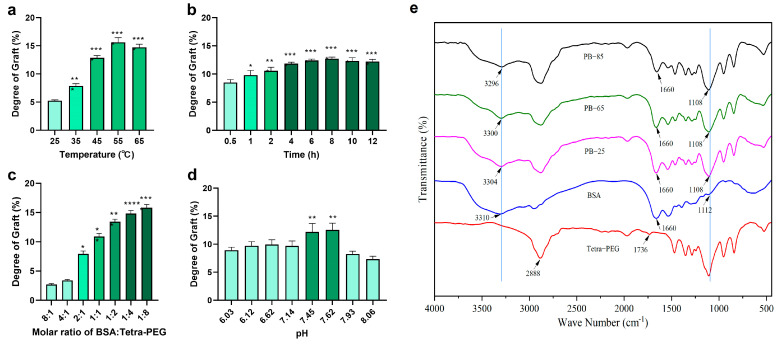
Graphs showing the grafting degree (GD) of BSA-PEG hydrogels under different conditions: (**a**) temperature, (**b**) time, (**c**) molar ratio, (**d**) pH, and (**e**) Fourier transform infrared spectroscopy (FTIR) spectra of BSA-PEG hydrogels at different temperatures. The symbols “*”, “**”, “***” and “****” represent the levels of statistical significance between groups, with the following meanings: *p* < 0.05 (significant difference), *p* < 0.01 (highly significant difference), *p* < 0.001 (very highly significant difference), and *p* < 0.0001 (extremely highly significant difference), respectively.

The reaction kinetics were greatly affected by temperature (Figure 2a). When the temperature was raised from 25 °C to 50 °C, the GD increased as well due to the increased motion of the molecules and reaction kinetics. At the same time, at 60 °C, GD decreased because BSA lost its structure as a result of thermal denaturation. These results imply that GD can be maximized within a temperature range of 25–50 °C without destroying protein stability [21].

The GD, which was substantially elevated within the first 6 h, plateaued at 12 h (Figure 2b). This outcome suggests a two-step process in which the first phase is somehow chemically controlled, because the initial formation of a Schiff base was fast, followed by a diffusion-limited step as the free amino groups of BSA became saturated [22]. The plateau indicates that almost all the available aldehyde groups on Tetra-PEG and the amino groups on BSA had reacted by 12 h. This meant that there was little increase in GD with further reaction times, strengthening the reaction equilibrium [23].

GD was largely affected by the molar ratio of BSA to the Tetra-PEG aldehyde groups (Figure 2c). As the molar ratio increased from 1:1 to 1:40, GD increased rapidly. Beyond this ratio, GD reached a plateau, suggesting that excess aldehyde groups had been produced and did not participate in additional crosslinking. This observed saturation at a 1:20 ratio is consistent with the stoichiometric balance between the reactive amine groups present per BSA molecule and the aldehyde groups present per Tetra-PEG molecule. Aldehyde groups exceeding this threshold are likely to be reacting with water or other non-specific components [24]. A steep increase in GD is observed with an increasing molar ratio, which subsequently plateaus at higher ratios. This trend suggests that at lower molar ratios, grafting efficiency is high due to the abundance of available reactive sites. However, as the molar ratio increases, the system approaches saturation, limiting further grafting. Similar observations have been reported in protein–polymer conjugation systems [25,26], where excess polymer leads to steric hindrance and reduced reactivity of the remaining functional groups. This reflects a balance between reactant availability and spatial constraints during the grafting process.

The results (Figure 2d) showed that GD was pH-dependent, with higher values at pH 7–9. At this pH range, the amine groups on BSA are nucleophilic enough to effectively react with the aldehyde groups on Tetra-PEG to efficiently form Schiff bases [27]. When pH is low (<7), the amine groups on BSA are protonated, which decreases their nucleophilicity, and hence the reaction rate is slower. In contrast, at higher pH (>9), while the amine groups are still reactive, the denaturation of protein or non-specific side reactions may inhibit GD.

The FTIR spectrum showed a peak at 1736 cm^−1^ due to the stretching vibration of the C=O bond of the aldehyde group and a peak at 2888 cm^−1^ reflecting C–H bond stretching in the aldehydes (Figure 2e), confirming the presence of active CHO groups in Tetra-PEG. The peaks on these spectra confirm that there are active CHO groups in Tetra-PEG. Peaks at 1536 cm^−1^ and 3310 cm^−1^ are attributed to the stretching vibration of N–H in amines along the BSA spectrum, illustrating numerous free amino units. In PB (Tetra-PEG-BSA) hydrogels, the absorption intensity at 3310 cm^−1^ dramatically decreased after being modified with Tetra-PEG, and the peaks were blue-shifted to 3304 cm^−1^, 3300 cm^−1^, and 3296 cm^−1^ with the increased degree of modification. This means that there are less free amino groups and major conformational alterations in the molecules, probably resulting from the hydrogen bond formations between Tetra-PEG and BSA. Moreover, on the side of BSA, the characteristic peak at 1112 cm^−1^ (C–N bond) was elevated post-modification, in which the peak shifted towards a shorter wavelength at 1108 cm^−1^ in the PB hydrogel, as shown by the auxiliary lines. The red shift observed in the modified protein indicates an increase in polarity; this indicates that the number of C–N single bonds has gone up, which implies more conformation was possible, likely through hydrogen bonding interactions [28].

**Figure 3:**
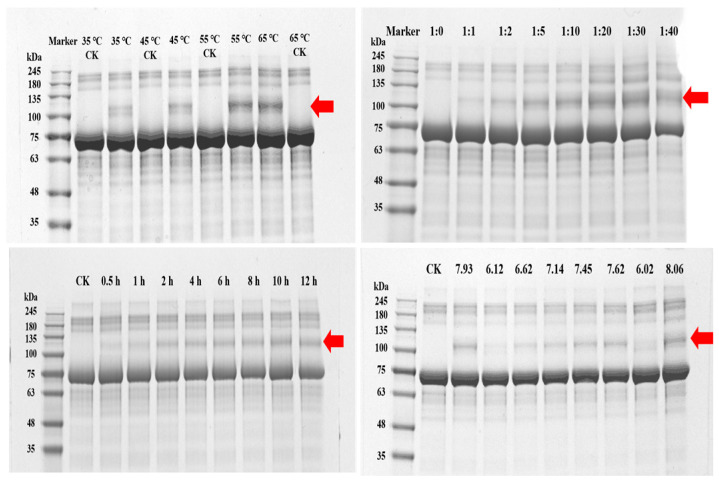
Graphs of SDS-PAGE images reflecting different temperature conditions, reaction times, molar ratios of reactants, and pH levels in the reaction system. (The red arrows highlight specific protein bands from the reactions, showing significant shifts in molecular weight under different conditions).

The results of the SDS-PAGE analyses (Figure 3) demonstrate that the molecular weights of the BSA-Tetra-PEG hydrogels were tested under different conditions. The molar ratio of BSA to Tetra-PEG (1:1 to 1:40) increased, and with increased modification, the band corresponding to unmodified BSA (66.5 kDa) decreased and higher molecular weight bands appeared (Figure 3). Temperature played a significant role, with bands for crosslinked products becoming more intense between 35 °C and 55 °C; however, these bands were modest with further increases to 65 °C, indicating protein denaturation at higher temperatures. Crosslinking was also additively affected by increasing reactions from 0.5 h to 12 h, where higher-molecular-weight bands became visually evident, further confirming these were time-dependent reactions. Crosslinking was most successful from pH 6.0 to 8.0, as evidenced by the elimination of lower-molecular-weight bands and the formation of larger aggregates [29].

These in-depth experiments show the relationship between experimental variables and the crosslinking efficiency of BSA-Tetra-PEG hydrogels. The maximum grafting conditions for BSA and Tetra-PEG were at a reaction time of 6–12 h, pH of 7–9, 25–50 °C, and at a molar ratio of 1:4. The FTIR and SDS-PAGE analyses validated the grafting and elucidated the molecular interactions between BSA and Tetra-PEG. These findings are a scaffold for the expeditious synthesis of protein-based hydrogels.

### 2.2. Intrinsic Fluorescence Spectroscopy

**Figure 4:**
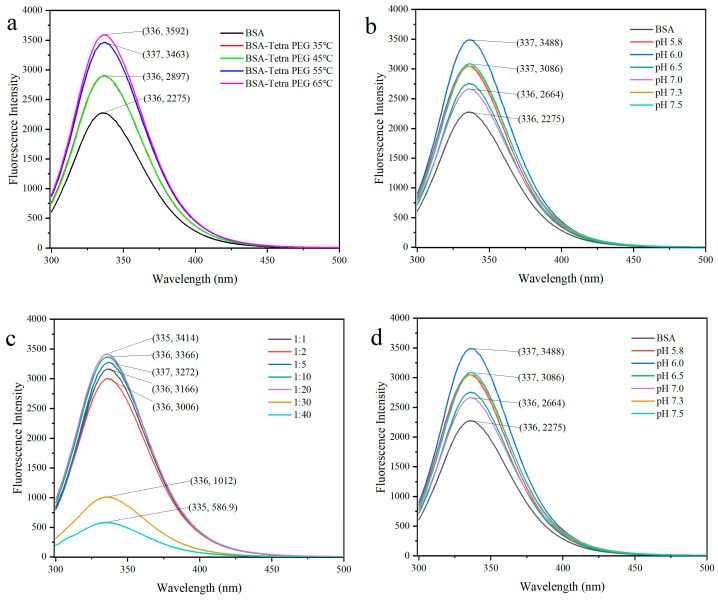
Fluorescence spectra of PEG-BSA mixed solution under different (**a**) temperature conditions, (**b**) reaction times, (**c**) molar ratios, and (**d**) pH levels in the reaction system.

These findings also are consistent with other studies indicating that Tetra-PEG modification leads to major conformational alterations in BSA, as shown by fluorescence quenching and peak shifts. The data here also underscore the sensitivity of intrinsic fluorescence to track protein structural dynamics during chemical modification [30]. The intrinsic fluorescence spectra of BSA, illustrated in Figure 4, clearly demonstrates significant alterations that occur based on the experimental arrangement and can offer insight into the dynamics of protein structure. In non-modified BSA, the native conformation of the protein is indicated by a peak at around 335–336 nm associated with the fluorescence characterization of tryptophan residues. After modification with Tetra-PEG, a marked increase in fluorescence intensity with a red shift in the position of the peaks indicates the increased exposure of tryptophan residues to a more polar environment, indicating that the hydrophobic regions inside the protein were exposed to the outside. These changes in fluorescence characteristics suggest that Tetra-PEG modification affects the fluorescence properties of BSA by altering its structure and exposure environment, further suggesting that intrinsic fluorescence can be an effective tool for tracking dynamic changes in protein structure. These findings are in agreement with other findings suggesting that Tetra-PEG modification is capable of inducing significant changes in protein conformation [31].

The reaction conditions had a significant impact on the degree of fluorescence quenching. The quenching was greater at higher Tetra-PEG concentrations (1:20 and 1:40 molar ratios), indicating extensive crosslinking and conformational re-arrangement. A slow decline in fluorescence intensity was detected over a reaction period of 12 h, corresponding to the progressive modification of and conformational change in both proteins. Maximal quenching was observed at pH 7–9, which indicates that conformational rearrangements are preferred in neutral to weakly basic conditions with successful Schiff base formation. Fluorescence quenching also increased with the addition of temperatures up to 50 °C; however, exceeding this threshold level will likely cause protein denaturation by compromising the structural integrity of BSA [32].

Fluorescence quenching and the shift in intensity peaks indicated that a substantial conformational change in BSA induced by Tetra-PEG modification had taken place. In addition, as intrinsic fluorescence is sensitive enough to reflect the structural dynamics of proteins, it can be applied as a tool to monitor these dynamics when proteins are chemically modified.

### 2.3. Macro- and Micromorphology

**Figure 5:**
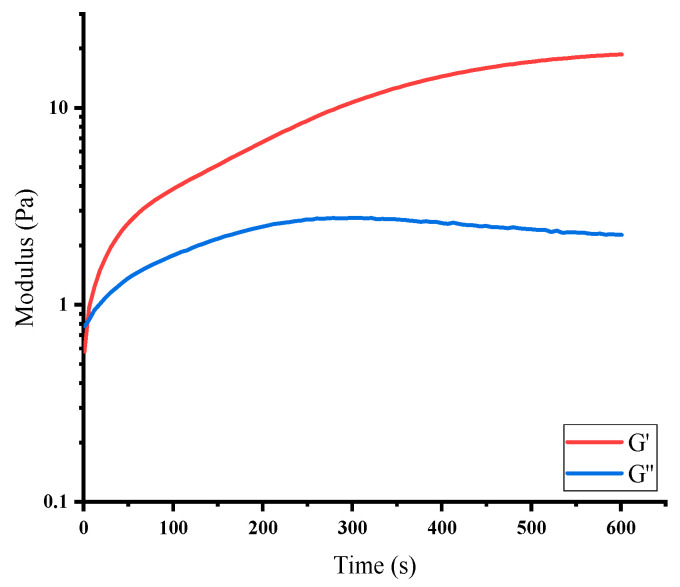
The trends of G′ and G″ over time for BSA and Tetra PEG-CHO.

Figure 5 presents the frequency-dependent rheological behavior of the hydrogels, illustrating the storage modulus (G′) and loss modulus (G″). The storage modulus (G′), which represents the elastic or solid-like response of the hydrogel, increases steadily with frequency and remains higher than the loss modulus (G″) across all measured frequencies. This dominance of G′ over G″ confirms that the hydrogel exhibits a predominantly elastic, solid-like behavior, consistent with the formation of a well-developed crosslinked network. Such viscoelastic behavior is typical for hydrogels with strong covalent crosslinking, as previously described for PEG-based and protein–polymer hydrogels. The observed increase in G′ with frequency also reflects the network’s capacity to resist deformation under applied stress, highlighting its mechanical stability and suitability for biomedical or structural applications.

These results demonstrate that the degree of grafting and the mechanical properties of the hydrogel are closely interconnected. The decline in GD at higher molar ratios aligns with the improved mechanical performance (higher G′), suggesting that increased crosslinking density strengthens the hydrogel network but reduces grafting efficiency due to limited available binding sites. Moreover, the solid-like behavior indicated by G′ > G″ supports the formation of a robust, elastic network, which is essential for applications requiring mechanical resilience, such as tissue engineering or drug delivery systems [10,33,34].

Table 1 compares the gelation times of hydrogels formed by different methods: Tetra-PEG-BSA, GLU-BSA, HT-BSA, and GDL-BSA gels. The Tetra-PEG-BSA gel exhibits the shortest gelation time (around 11 s), indicating highly efficient crosslinking driven by the rapid Schiff base reaction between the aldehyde groups of Tetra-PEG-CHO and the amine groups of BSA. Such fast gelation is advantageous in applications requiring rapid hydrogel formation, such as injectable systems for tissue engineering or drug delivery [33]. In contrast, the GLU-BSA gel requires a significantly longer gelation time, likely due to the slower diffusion and lower reactivity of glutaraldehyde with BSA. The prolonged crosslinking process observed here is consistent with previous studies, where aldehyde-based crosslinking often results in delayed gel formation [35]. The HT-BSA gel shows a moderately fast gelation time (around 17 s), attributed to heat-induced protein denaturation and aggregation, which facilitates network formation but requires thermal energy to initiate the process [36]. Conversely, the GDL-BSA gel demonstrates the slowest gelation time (around 6 h) due to the gradual acidification caused by GDL, which lowers the pH over an extended period and promotes slow protein assembly [37]. These differences emphasize the critical role of the gelation mechanism in determining hydrogel formation speed, with chemical crosslinking methods, such as Tetra-PEG-BSA, offering superior efficiency compared to thermal or pH-triggered approaches.

The transmittance values at 600 nm, also presented in Table 1, provide insights into the optical clarity and structural homogeneity of the hydrogels. The Tetra-PEG-BSA gel achieves the highest transmittance (92.92%), suggesting the formation of a highly uniform and transparent network. This can be attributed to the rapid and well-distributed chemical crosslinking facilitated by the Tetra-PEG system, which minimizes phase separation and aggregation during gelation [38]. In comparison, the GLU-BSA gel exhibits moderate transmittance (72.48%), likely due to heterogeneous crosslinking and localized gelation induced by glutaraldehyde. Such non-uniformity is a common issue in aldehyde-mediated systems, where uneven crosslinking leads to turbidity and reduced clarity. Both the HT-BSA gel and GDL-BSA gel display extremely low transmittance values (4.80% and 3.13%, respectively), indicating highly turbid and inhomogeneous networks. In the HT-BSA gel, heat-induced protein denaturation causes aggregation and phase separation, resulting in significant opacity [39]. Similarly, the slow pH reduction in the GDL-BSA gel promotes uncontrolled protein assembly, leading to a poorly organized and opaque structure [40].

These results underscore the superior performance of Tetra-PEG-BSA gels, which combine rapid gelation with high transparency, making them ideal for applications requiring optical clarity and precise network formation, such as bioimaging scaffolds or optical sensors. In contrast, the limited transparency and slow gelation observed in the other methods highlight their drawbacks for applications demanding rapid, uniform, and clear hydrogels.

**Figure 6:**
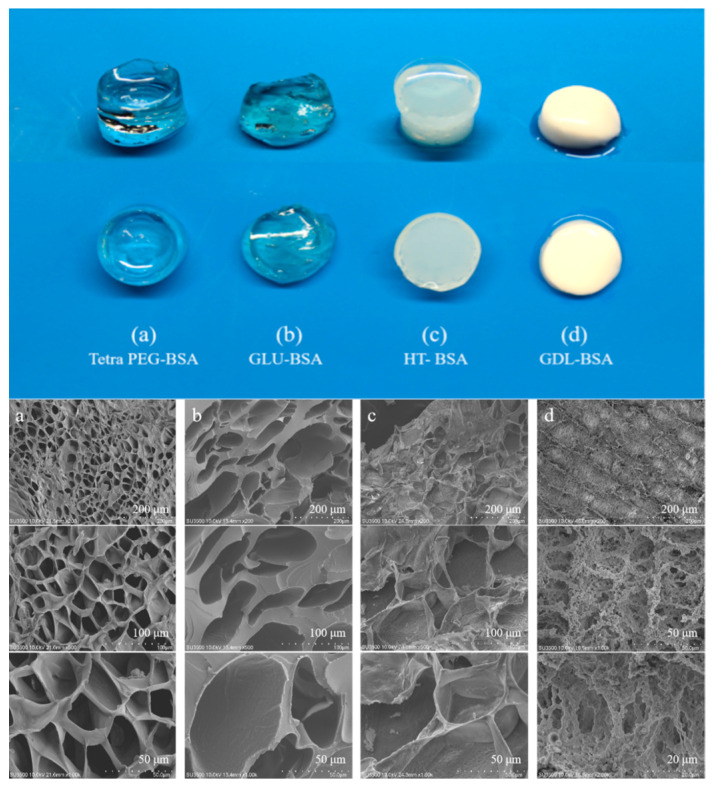
Macroscopic appearance and microscopic morphology of hydrogels: (**a**) Tetra-PEG-BSA, (**b**) GLU-BSA, (**c**) HT-BSA, and (**d**) GDL-BSA. Scanning electron microscopy (SEM) images at varying magnifications reveal the internal microstructures of each hydrogel.

Figure 6 presents a comprehensive comparison of the macroscopic appearance and microscopic morphology of Tetra-PEG-BSA, GLU-BSA, HT-BSA, and GDL-BSA hydrogels, highlighting structural variations at both the macro- and micro-levels. Horizontally, across all samples, the macroscopic appearance reveals stark differences: Tetra-PEG-BSA hydrogels are highly transparent and homogeneous, whereas GLU-BSA hydrogels show partial transparency, and both HT-BSA and GDL-BSA hydrogels appear opaque and turbid. These trends are consistent with the SEM results, where Tetra-PEG-BSA hydrogels exhibit a highly interconnected and uniform porous network with evenly distributed pores ranging from 50 to 200 µm. In contrast, GLU-BSA shows larger, irregular pores with uneven walls, HT-BSA reveals dense aggregated regions with minimal pore connectivity, and GDL-BSA presents a compact, disordered structure with poorly connected micropores. These observations confirm that rapid and efficient crosslinking, as seen in Tetra-PEG-BSA, promotes homogeneous network formation, while slower or thermally induced gelation mechanisms result in inhomogeneous and turbid structures [41].

Additionally, examining each sample individually reveals clear links between gelation time, transparency, and internal morphology. Tetra-PEG-BSA gels demonstrate superior performance, with the fastest gelation time (around 11 s), highest transparency (92.92%), and a well-organized porous microstructure. These results stem from the efficient Schiff base reaction between aldehyde and amine groups, which minimizes phase separation and promotes uniform network formation [36]. GLU-BSA gels, in contrast, require significantly longer gelation time and exhibit moderate transparency (72.48%). This slower glutaraldehyde-mediated crosslinking leads to uneven network development, resulting in irregular pore structures and reduced optical clarity [42]. HT-BSA gels, formed via heat-induced denaturation and aggregation, gel quickly but show minimal transparency (4.80%) and dense, disordered microstructures due to uncontrolled protein aggregation. Lastly, GDL-BSA gels, with their extremely slow gelation time, display the lowest transparency (3.13%) and compact, highly disorganized microstructures caused by gradual acidification and uncontrolled protein assembly [42,43].

In conclusion, the Tetra-PEG-BSA hydrogels outperform the others in terms of transparency, structural homogeneity, and gelation efficiency, making them suitable for applications requiring precision and optical clarity, such as tissue engineering or drug delivery systems. The differences observed in the GLU-BSA, HT-BSA, and GDL-BSA hydrogels emphasize the impact of gelation mechanisms on network organization, optical properties, and functional performance. Optimizing gelation strategies is therefore critical for tailoring hydrogel properties to meet specific application requirements.

**Figure 7:**
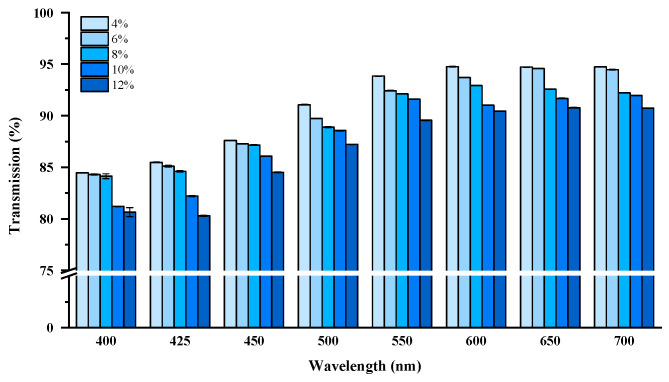
Light transmittance of Tetra-BSA hydrogel in different concentrations of PEG solution: Tetra-PEG-BSA hydrogels induced by different Tetra-PEG concentrations of 4%, 6%, 8%, 10%, and 12%.

Optical transmittance of the hydrogels was investigated using a UV spectrophotometer at a wavelength range of 400–700 nm, with distilled water as the calibrator. The hydrogels were cut into slices with a thickness of 2 mm and adjusted to the inner wall of glass cuvettes for the interrogation of the light beam across the biomass sample. As shown in Figure 7, transmittance generally increased with longer wavelengths, indicating that the hydrogels absorbed or scattered less light in the red region compared to the blue region of the visible spectrum. Sample 5 (12%) exhibited the lowest transmittance across all measured wavelengths, suggesting a denser network or increased particulate presence that reduces optical clarity. Sample 1 (4%) demonstrated the highest transmittance, indicating improved optical clarity. These differences became more pronounced at longer wavelengths (600–700 nm), likely due to variations in structural properties such as polymer concentration, degree of grafting, or crosslinking density [42,44]. Hydrogels with higher transmittance, such as S1, may possess fewer scattering centers, making them suitable for applications requiring optical transparency, such as contact lenses, optical sensors, or biomedical devices [43]. Conversely, hydrogels with lower transmittance, like S5, reflect network irregularities or increased microstructural opacity.

### 2.4. Swelling Radio

Swelling is a phenomenon where a polymer absorbs solvent and expands in volume, and its extent is closely linked to the network structure and crosslinking efficiency of hydrogels [45]. In this study, four types of hydrogels—Tetra-PEG-BSA, GLU-BSA, HT-BSA, and GDL-BSA—were tested in various solutions to investigate their swelling behavior. Across all solutions, Tetra-PEG-BSA gels consistently demonstrate the highest swelling ratio. This superior performance can be attributed to their uniform and well-interconnected network structure, which facilitates efficient solvent absorption [46]. In contrast, the swelling ratios of GLU-BSA, HT-BSA, and GDL-BSA gels are significantly lower. GLU-BSA gels show moderate swelling due to slower and less uniform crosslinking, while HT-BSA and GDL-BSA gels exhibit minimal swelling, reflecting their dense and disordered microstructures that restrict solvent penetration. The differences in swelling behavior among the hydrogels align with the SEM analysis, where Tetra-PEG-BSA gels revealed a highly porous network, while the other gels displayed irregular or compact morphologies. Thus, the swelling capacity is strongly dependent on the uniformity and quality of the crosslinked network [46,47].

**Figure 8:**
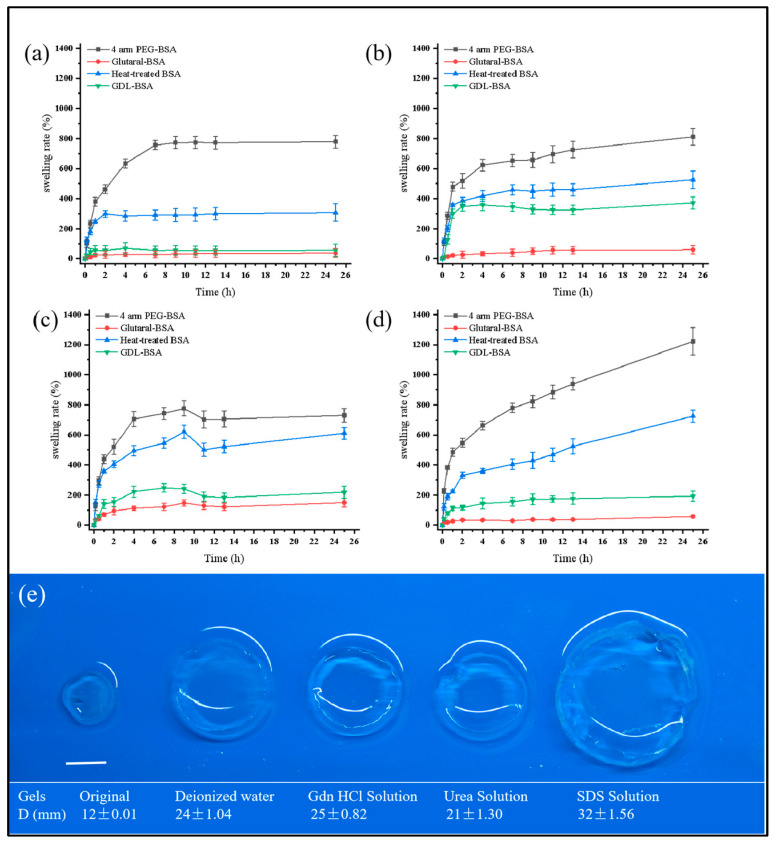
The swelling properties of four hydrogels in (**a**) water, (**b**) 4 mol/L Gdn HCl solution, (**c**) 8 mol/L urea solution, and (**d**) 20 mmol/L SDS solution. (**e**) The size of the hydrogels before swelling and after swelling equilibrium in different solutions. (The scale bar is 10 mm).

The swelling behavior of the hydrogels reveals clear differences across different hydrogels and in different solutions (Figure 8). Tetra-PEG-BSA gels consistently exhibit the highest swelling ratios due to their uniform, highly crosslinked, and porous network structure, as observed in the SEM analysis in Figure 6. This superior performance is maintained even in denaturing solutions such as guanidine hydrochloride, urea, and SDS, reflecting the robustness and stability of their crosslinked network. In contrast, GLU-BSA, HT-BSA, and GDL-BSA gels show limited swelling capacity, especially under chemical denaturation conditions, due to their irregular or compact microstructures and weaker crosslinking efficiency. These results highlight the importance of optimizing crosslinking strategies to improve the network uniformity and chemical stability of hydrogels, making them suitable for applications requiring high swelling capacity and structural robustness under various environmental conditions [42,44,46].

These observations serve to validate that uniformity in networks and crosslinking efficiency are important factors in controlling the swelling capacity and durability of hydrogels. Not only do these observations validate Tetra-PEG-BSA gels’ high performance, but through the integration of SEM and swelling data, it is apparent that uniformity in structures and effective crosslinking have important roles in controlling overall performance in hydrogels.

### 2.5. Mechanical Properties

**Figure 9:**
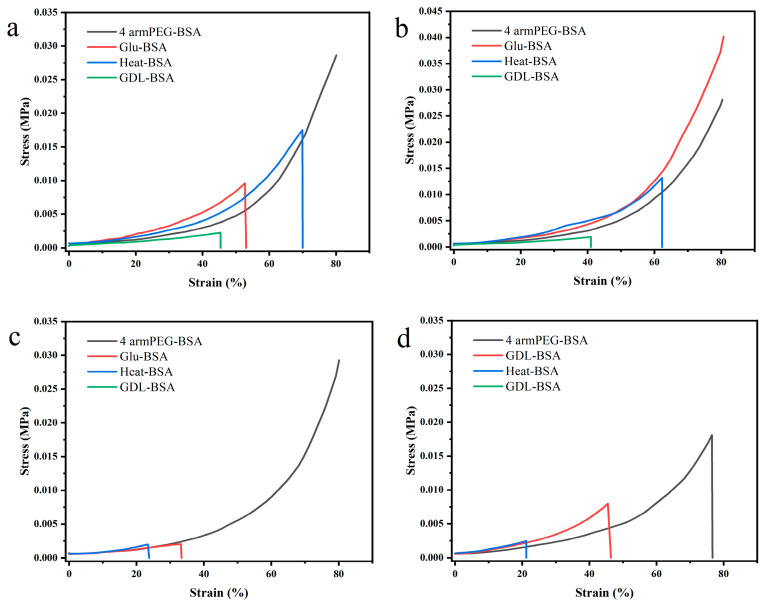
Stress–strain curves of Tetra-PEG-BSA, GLU-BSA, HT-BSA, and GDL-BSA hydrogels after swelling in (**a**) water, (**b**) 4 mol/L Gdn HCl solution, (**c**) 8 mol/L urea solution, and (**d**) 20 mmol/L SDS solution.

From the stress–strain curves (Figure 9), it is evident that the mechanical properties of the four types of gels varied significantly when swollen in different media, namely water, 4 mol/L guanidine hydrochloride (Gdn HCl) solution, 8 mol/L urea solution, and 20 mmol/L SDS solution. PEG-BSA gels demonstrated exceptional elasticity and toughness when swollen to equilibrium in water, Gdn HCl, and urea solutions. Even at 80% deformation, the gels remained unbroken, indicating the robustness and stability of the PEG-BSA network structure. This can be attributed to the effective crosslinking between PEG and BSA, which provides sufficient structural integrity to withstand external forces, even in partially denaturing environments [48,49]. In contrast, GDL-BSA gels lost their gel structure in both urea and SDS solutions, becoming dispersions rather than cohesive gels. This result suggests significant structural damage caused by the denaturation agents, which disrupt the ionic and covalent interactions that stabilize the gel matrix [50]. Protein denaturation solutions, such as high concentrations of Gdn HCl, urea, and SDS, are known to disrupt hydrogen bonds and reduce hydrophobic interactions. These effects lead to varying degrees of protein denaturation, particularly in Gdn HCl and urea solutions, where globular proteins typically assume an irregular, unfolded conformation. Consequently, gel strength was significantly reduced, and in some cases, the gels transitioned into non-gel dispersions [44].

The superior resistance of PEG-BSA gels in denaturing environments may be due to the uniform structure between protein molecules and PEG chains, which prevents the complete disintegration of the gel network. This highlights the critical role of crosslinking agents and molecular architecture in maintaining mechanical stability [44,51].

As summarized in Table 2, the maximum gel strength values varied depending on the gel type and swelling solution: GLU-BSA gels exhibited the highest strength in Gdn HCl solution (4.60 ± 0.05 N), suggesting strong crosslinking that withstands denaturation conditions. However, this high gel strength is likely a result of more rigid, less flexible networks, which may compromise elasticity. PEG-BSA gels maintained relatively high strength across the water (3.16 ± 0.66 N), Gdn HCl (3.43 ± 0.20 N), and urea solutions (3.26 ± 0.26 N), highlighting their consistent mechanical performance under diverse conditions [51]. This indicates that PEG crosslinking provides both flexibility and strength, contributing to the overall resilience of the gel. In contrast, GDL-BSA gels did not form a stable gel structure after swelling in urea and SDS solutions, emphasizing their susceptibility to network degradation in denaturing environments. This indicates that GDL-induced gelation may rely more on ionic and hydrogen bonds, which are easily disrupted by denaturing agents [52].

#### Compressive Properties Analysis

The compressive properties of the four hydrogels are compared in Table 3. The key findings include that Tetra PEG-BSA gels exhibited the highest fracture strain (84.12 ± 0.76%) and rupture stress (28.64 ± 1.21 kPa), along with the lowest elastic modulus (14.7 ± 2.4 Pa) and the highest fracture energy (468.0 ± 34.9 kJ/m^3^). These results highlight that PEG-BSA gels showed superior elasticity, toughness, and energy absorption ability, in comparison to the other three types. The combination of high strain and low modulus suggests that PEG-BSA gels can absorb large amounts of energy under deformation without structural failure [1,51]. GLU-BSA gels displayed moderate mechanical performance, with a fracture strain of 52.70 ± 0.58% and a rupture stress of 9.57 ± 0.32 kPa. The relatively higher modulus (44.0 ± 4.5 Pa) indicates a stiffer structure, which limits the gel’s ability to undergo large deformations but provides some resistance to external stress. HT-BSA gels exhibited better compressive properties than GDL-BSA gels, achieving a fracture strain of 69.92 ± 1.17% and a rupture stress of 17.53 ± 0.54 kPa. This demonstrates moderate elasticity and toughness, likely due to thermal-induced structural stabilization. GDL-BSA gels had the weakest mechanical properties, with the lowest fracture strain (45.39 ± 0.54%) and rupture stress (2.23 ± 0.30 kPa), as well as minimal energy dissipation capacity (50.3 ± 2.6 kJ/m^3^). This result can be attributed to the less stable and easily disrupted network formed through GDL-induced gelation [51].

The results collectively highlight the superior mechanical performance of Tetra PEG-BSA gels, which achieved the highest elasticity, toughness, and energy dissipation. This exceptional performance can be attributed to several factors. First, the four-armed PEG structure provides effective crosslinking with BSA, creating a stable yet flexible network capable of withstanding deformation and stress without fracturing, even under extreme conditions such as swelling in denaturing agents [51]. Second, the combination of PEG and BSA ensures a balance between hydrogen bonds and hydrophobic interactions, which enhances network integrity and stability in partially denaturing environments [53]. The high fracture energy of Tetra PEG-BSA gels further indicates excellent energy dissipation, making them particularly suitable for applications requiring mechanical robustness, such as tissue engineering and load-bearing hydrogels. In comparison, GLU-BSA and HT-BSA gels, despite demonstrating moderate performance, lack the elasticity and toughness of Tetra PEG-BSA gels due to their stiffer or less efficient crosslinking networks. Meanwhile, GDL-BSA gels exhibit significant susceptibility to denaturation, making them unsuitable for environments with strong denaturing agents. Overall, the mechanical superiority of Tetra PEG-BSA gels establishes them as promising candidates for advanced applications that demand a combination of elasticity, toughness, and structural stability [51].

The swelling behavior of each hydrogel was further analyzed in four solutions—water, guanidine hydrochloride, urea, and SDS—providing insights into their structural stability under different denaturation conditions. In the water, all hydrogels exhibit their maximum swelling ratios, with Tetra-PEG-BSA gels achieving the highest values due to their hydrophilic and well-organized network [47]. GLU-BSA, HT-BSA, and GDL-BSA gels show reduced swelling in comparison, primarily because of their irregular crosslinking and less porous structures [42]. Water, as a neutral solvent, highlights the intrinsic swelling capacity of the hydrogels without inducing network disruption. In guanidine hydrochloride, a strong chaotropic agent that disrupts hydrogen bonds and hydrophobic interactions, the swelling ratios of all hydrogels decrease compared to water. The Tetra-PEG-BSA gels still exhibit relatively high swelling, suggesting their network stability and resistance to chemical denaturation. In contrast, the swelling of GLU-BSA, HT-BSA, and GDL-BSA gels is significantly limited, reflecting their weaker structures, which are more prone to collapse under chemical stress. In urea solution, a denaturing agent that reduces hydrophobic interactions, a similar trend is observed. Tetra-PEG-BSA gels maintain a higher swelling ratio, indicating the robustness of their crosslinked network. However, GLU-BSA, HT-BSA, and GDL-BSA gels show further reduced swelling, suggesting that urea destabilizes their already less organized networks [46]. The consistent performance of Tetra-PEG-BSA highlights its superior chemical resistance. In SDS solution, an anionic surfactant that disrupts hydrophobic interactions and induces protein unfolding, the swelling ratios of all hydrogels decrease further. While Tetra-PEG-BSA gels maintain the highest swelling capacity, the performance of GLU-BSA, HT-BSA, and GDL-BSA gels remains minimal, indicating significant structural disruption. The lower swelling ratios in SDS highlight the vulnerability of weakly crosslinked and disordered networks to surfactant-induced stress [52,54].

The combination of swelling properties and light transmittance measurements highlights a key relationship between network integrity and environmental adaptability. Specifically in terms of swelling behavior and optical transmittance, Tetra-PEG-BSA hydrogels consistently exhibit superior swelling performance due to their highly uniform, interconnected networks, which allow efficient solvent absorption without significant structural collapse. At lower polymer concentrations or under neutral conditions, hydrogels retain higher transmittance due to the minimal presence of scattering centers and uniform pore distribution. However, a gradual decline in transmittance at higher PEG concentrations reflects network compression under osmotic stress, reducing optical clarity. This correlation suggests that hydrogel transparency and swelling capacity are strongly influenced by crosslinking density, uniformity, and structural stability [43,51].

Despite the excellent optical and swelling performance of Tetra-PEG-BSA hydrogels, their reduced transmittance at higher osmotic pressures raises concerns about network collapse and structural instability under concentrated environments. For practical applications requiring both high swelling and optical transparency, further optimization is needed, mainly in terms of enhancing crosslinking density and incorporating stabilizing agents. Increasing the degree of crosslinking could stabilize the network, reducing structural collapse under osmotic stress [48,51,55].

### 2.6. Cell Viability Assay In Vitro

**Figure 10:**
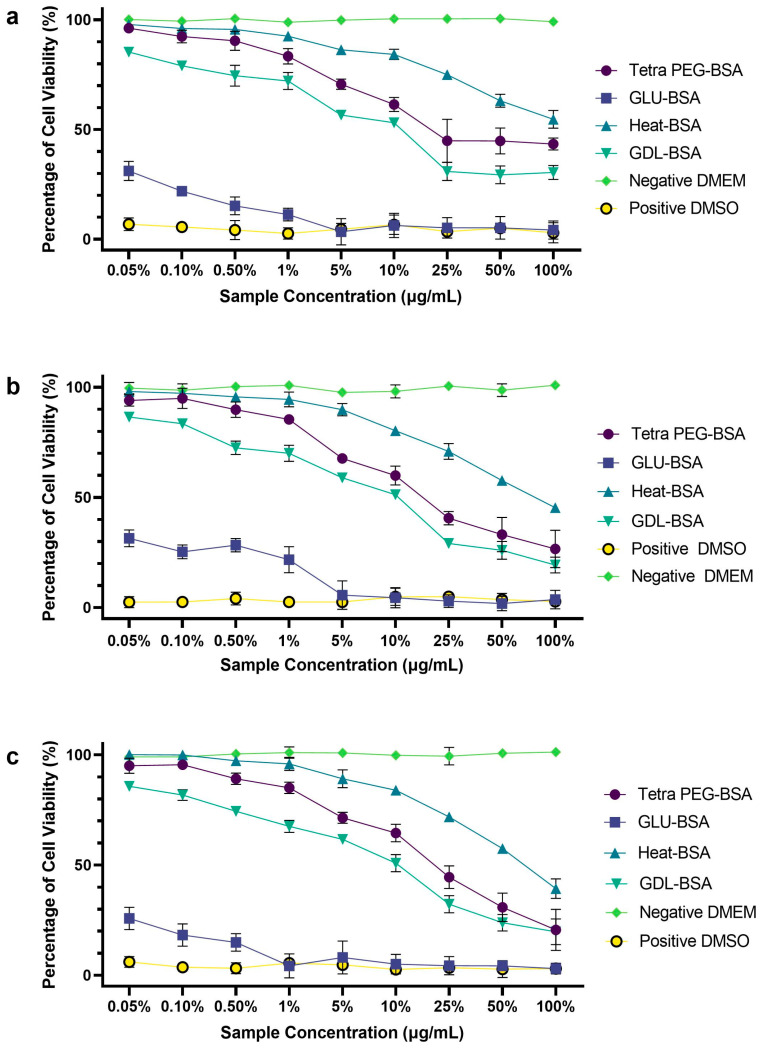
Cell viability of different hydrogels after (**a**) 24 h, (**b**) 48 h, and (**c**) 72 h exposures, determined by the MTT assay. Data are expressed as a percentage of control mean ± SD of three independent experiments.

The cell viability of Tetra PEG-BSA, Glu-BSA, Heat-BSA, and GDL-BSA hydrogels at different concentrations (0.05–100%) was assessed using the MTT assay over 24, 48, and 72 h, as illustrated in Figure 10. The findings indicate that Tetra PEG-BSA and Heat-BSA hydrogels demonstrate superior biocompatibility compared to Glu-BSA and GDL-BSA, with cell viability decreasing in a concentration- and time-dependent manner.

At 24, 48, and 72 h, exposure to 100% Tetra PEG-BSA resulted in cell viability levels of 43.38%, 26.60%, and 25.59%, respectively. Meanwhile, 100% Heat-BSA maintained viability at 54.64%, 45.36%, and 39.27%, indicating minimal levels of cytotoxicity even at high concentrations. In contrast, Glu-BSA hydrogels exhibited significant cytotoxic effects, with viability decreasing to 5.52%, 4.16%, and 3.01% at 100% extract concentration, suggesting extensive cell death at higher doses. GDL-BSA showed moderate cytotoxicity, with viability measured at 30.41%, 19.33%, and 16.39% at 100% concentration, reflecting a steady decline over time.

At lower concentrations (0.05–1%), Tetra PEG-BSA and Heat-BSA maintained cell viability above 80% at 24 h, while GDL-BSA remained above 70% in this range. However, Glu-BSA displayed a sharp decrease, with viability dropping below 30% at concentrations exceeding 0.5%, indicating that only minimal doses are safe for maintaining cell viability. The noticeable reduction in viability at 72 h for all hydrogels suggests that prolonged exposure increases cytotoxicity, underscoring the importance of time-dependent effects on hydrogel biocompatibility.

The enhanced biocompatibility of PEG-modified and heat-treated hydrogels aligns with previous studies, which suggest that PEGylation improves hydrophilicity, minimizes protein adsorption, and reduces immune responses [44,56]. The superior performance of Heat-BSA hydrogels further indicates that controlled thermal processing helps preserve protein integrity, thereby lowering toxicity [56]. Conversely, the high cytotoxicity of Glu-BSA hydrogels is consistent with findings that glutaraldehyde crosslinking introduces aldehyde residues, triggering oxidative stress and apoptosis [57]. GDL-BSA hydrogels display intermediate cytotoxicity, likely due to the slow release of gluconic acid, which may disrupt cellular pH balance and metabolic activity [58].

These findings suggest that Tetra PEG-BSA and Heat-BSA hold promise as biomaterials for applications such as tissue engineering, drug delivery, and 3D bioprinting due to their low cytotoxicity and stable performance over time. However, Glu-BSA hydrogels may require alternative crosslinking methods (like EDC/NHS) to improve biocompatibility [59], while GDL-BSA formulations could benefit from pH-buffering strategies to counteract potential acidity-induced cytotoxicity. The results reinforce previous research highlighting the crucial role of material physicochemical properties—such as size, crosslinking chemistry, and degradation behavior—in determining cytotoxicity outcomes. Further studies on long-term cellular interactions, biodegradability, and in vivo compatibility will be necessary to validate these hydrogels for clinical application [60,61].

## 3. Conclusions

Herein, we present a novel Schiff base-crosslinked hydrogel formed from the reaction of Tetra-armed polyethylene glycol with aldehyde end groups (Tetra-PEG-CHO) and bovine serum albumin (BSA) under alkaline conditions. The Tetra-PEG-BSA hydrogel exhibited excellent tear gelation kinetics, with rapid gelation at 11 s, whereas the rapid gelation times of the GLU-BSA, HT-BSA, and GDL-BSA hydrogels were significantly longer. The Tetra-PEG-BSA-based hydrogel showed relatively superior optical properties, with a transmittance of 92.92% at 600 nm, indicating a uniform and clear crosslinked network structure.

Mechanical tests showed excellent performance with a fracture strain of 84.12 ± 0.76%, rupture stress of 28.64 ± 1.21 kPa, and fracture energy of 468.0 ± 34.9 kJ·m^−3^, representing better values than the other three kinds of hydrogels. We validated the hydrogel’s structural stability and mechanical integrity in harsh environments, such as solutions of 4 M guanidine hydrochloride, 8 M urea, and 20 mmol/L SDS, with rheological and swelling studies.

Fluorescence quenching studies showed that the reorganization of protein conformation is very much dependent on the reaction conditions, and the best crosslinking and quenching were achieved at pH 7–9, 25–50 °C, a 12 h reaction time, and a BSA–Tetra-PEG molar ratio of 1:4. Efficient Schiff base formation and minimal protein denaturation were verified by FTIR and SDS-PAGE.

Biocompatibility was confirmed by an MTT cytotoxicity assay using MCF-7 cells, where cell viability was >80% at lower concentrations, rendering it compatible for biomedical applications. Tetra-PEG-BSA, owing to its biocompatibility, mechanical strength, and optical transparency, holds great potential for tissue engineering, drug delivery, bioimaging, and 3D printing.

In conclusion, the Tetra-PEG-BSA hydrogel is a clear, stable, and biocompatible hydrogel with excellent mechanical strength, and it shows excellent potential for various applications. Crosslinking density optimization, stabilizer addition, and functional modifications can further tune it for various environments.

## 4. Materials and Methods

### 4.1. Materials

Tetra-PEG (10 kDa) was procured from Meiluo Technology Ltd. (Shenzhen, China). It can be obtained through various methods, including nucleophilic addition reactions, ring-opening reactions of ethylene oxide with aldehyde reagents, and the oxidation of hydroxyl groups using oxidizing agents [24,62]. Bovine serum albumin V (BSA V, 98%), guanidine hydrochloride, urea, and SDS were obtained from Solarbio (Beijing, China). Dithiothreitol (DTT), glutaraldehyde, and D-(+)-gluconic acid δ-lactone (GDL) were obtained from Aladdin Industrial Corporation (Shanghai, China). HCl and NaOH were of analytical grade. No further purification of the materials was required; they were used as received.

### 4.2. Hydrogel Preparation and Gelation Time

The BSA stock solution (264 mg/mL) and Tetra-PEG-CHO (Mw 10,000 Da) solution were dissolved in PBS (10 mM, pH 7.0) solvent, respectively. Denaturing agent: The BSA solution was added to dithiothreitol (DTT) at a ratio of 1:66 (*w*/*w*) and stirred for 4 h. The Tetra-PEG-BSA hydrogel was formed by adding a solution of 0.5 mL BSA, acidified with 0.25 M HCl, to 10 (mL) Tetra-PEG-CHO at a concentration of 200 mg/mL. The resulting mixture was alkalized to a final concentration of 0.5 M NaOH solution. After approximately 10 s, the Tetra-PEG-BSA hydrogel formed. GDL was mixed with BSA stock based on a 1.5:100 (*w*/*w*) ratio and incubated in a water bath at 45 °C for 1 h to obtain a GDL-BSA hydrogel. HT-BSA hydrogel: BSA stock solution was incubated at 95 °C for 10 min. For GLU-BSA hydrogel, a 25% solution of glutaraldehyde was added to the BSA stock solution and kept overnight for reaction. A cylindrical mold is specific for this type of polymerization and a height of 0.6 mm and a diameter of 0.85 mm were utilized for all BSA hydrogels. Full mixing of all the components starts the measurement of the gelation time by the inversion method.

Gelation was established when the turned upside-down reactor maintained its shape without any movement of fluid for 30 s, with reversal every 30 min when gel time exceeded 1 h. This method provides a simple way to monitor the progress of gelation. The gelation time of the PEG-BSA hydrogel was further confirmed with a Discovery HR-3 rheometer (TA Instruments, New Castle, DE, USA) using a 40 mm parallel plate fixture at a 1000 μm gap and 25 °C.

### 4.3. Detection of Grafting Degree

The trinitrobenzene sulfonic acid (TNBS) method was used to ascertain the graft degree. An amount of 1 mL of protein solution (1 mg/mL) was mixed with TNBS solution (25 μL, 0.03 mol/L). The absorbance was measured at 420 nm (A_1_) after a 30 min incubation period at room temperature. The modified protein solution was prepared by mixing 1 mL of the modified protein solution (1 mg/mL) with 25 μL of trinitrobenzene sulfonic acid solution (0.03 mol/L) and reacting at room temperature for 30 min; then, the absorbance was measured at 420 nm (marked the result A_0_).

Graft degree (GD, %) was computed according to the following equation:

$$\mathrm{GD}\left(\%\right)=\frac{A_{0}-A_{1}}{A_{0}}\times 100\%$$

### 4.4. FTIR Spectroscopy

FTIR spectroscopy was used to study the molecular interactions in various samples. To reduce molecular mobility and weaken the hydrogen bond network between the water molecules and other components, the samples were lyophilized before FTIR measurement. We mixed the samples (2.0 mg) with 198.0 mg of potassium bromide (KBr) powder thoroughly, and then the mixture was pressed into uniform, transparent thin slices. A PerkinElmer Spectrum 100 FTIR Spectrometer with a resolution of 4 cm−1 was used to obtain Fourier-transform infrared (FTIR) spectra (PerkinElmer, Buckinghamshire, UK). The wavelength range was set to 400–4000 cm^−1^ and there were 64 scans for each sample. All spectra were automatically smoothed and baseline-corrected in Origin software version 2018.

### 4.5. SDS-PAGE

The electrophoretic bands produced in the SDS-PAGE tests were examined to study the formation of the conjugate. A dilute of the crosslinked product (3 mg/mL) was mixed with a loading buffer (4:1 volume ratio). The mixture was boiling-bath-heated for 10 min, and then centrifuged and separated with 12% polyacrylamide gel. Stacking (80 V) and separation (110 V) electrophoresis conditions were applied.

The gel samples were stained in a solution of 6.8% (*v*/*v*) acetic acid, 50% (*v*/*v*) methanol, and 0.01% (*w*/*v*) Coomassie Brilliant Blue R-250, followed by destaining in a solution of 7.5% (*v*/*v*) acetic acid and 10% Coomassie Brilliant Blue R-250. Staining and destaining were performed in the dark. Destained gels were photographed in transmitted white light and stored for the next analysis.

### 4.6. Intrinsic Fluorescence Spectra

The samples were diluted in distilled water to obtain a concentration of 0.2 mg/mL. Fluorescence scanning was conducted with an F-7000 fluorescence spectrophotometer (Hitachi, Tokyo, Japan). The excitation wavelength was kept constant at 280 nm. The excitation slit was 3 mm and the emission slit was 5 mm. Moreover, the scan range was 300–500 nm.

### 4.7. Transmittance Measurement

The hydrogels were then cut into 2 mm slices. They were subsequently glued to the internal walls of glass cuvettes. This allowed the light beam to pass through them. Using UV spectrophotometry, the transmittance was measured at 400, 425, 450, 500, 550, 600, 650, and 700 nm. Calibration was performed using distillate water.

### 4.8. Scanning Electron Microscopy (SEM)

This step aimed to study the microstructure of the 4 types of BSA hydrogels. First, the freeze-dried samples were fixed onto copper plates and sputter-coated with gold for 300 s. Images were made and captured with a field-emission scanning electron microscope (FE-SEM, HITACHI-SU3500, Tokyo, Japan). The voltage was an accelerating voltage of 10 kV and different magnifications were used, ranging from 200× to 1000×.

### 4.9. Determination of Swelling Ratio

The swelling ratio of the hydrogels was determined using a standard gravimetric method. Four types of BSA hydrogels were dried at 50 °C for 24 h. First, the mass of the fully dried gels was recorded. Then, the samples were immersed in deionized water (pH 7.0), 4 M guanidine hydrochloride, 8 M urea, and 20 mmol/L SDS, respectively. Subsequently, each gel was separated from the respective solutions after 10 min, 30 min, 1 h, 2 h, 4 h, 7 h, 9 h, 11 h, 13 h, and 25 h respectively. In addition, any excess surface water was carefully removed with filter paper. The gel weights were measured to the equilibrium.

The swelling ratio (SR) and equilibrium water content (EWC) were calculated using the following equations:

$$SR\left(100\%\right)=\frac{\left(m_{t}-m_{0}\right)}{m_{0}}\times 100\%$$

$$\mathrm{EWC}\left(100\%\right)=\frac{\left(m_{e}-m_{0}\right)}{m_{0}}\times 100\%$$

where m_0_ represents the initial dry weight of the gel at room temperature (g), denoting the weight of the gel after being removed from the solution and gently wiped at time t (g), and I is the weight of the gel at the equilibrium stage (g).

### 4.10. Mechanical Properties

Mechanical properties, a key feature in evaluating BSA gels, were tested using a compression program with a universal texture analyzer equipped with a 250 N force sensor (TMS-Pro, Food Technology Corporation, Sterling, VA, USA). Cylindrical, swollen hydrogel samples (Φ 8.5 mm × 6 mm) were prepared for testing. The starting force was set to 0.05 N, the compression rate was 20 mm/min, and the probe lift height was 10 mm. The samples were compressed to a strain of 80%, and stress–strain curves were recorded for all BSA hydrogel samples.

The compressive stress (σ) was calculated as follows:

$$\sigma_{c}=\frac{F}{A_{0}}$$

where F is the applied force (N) and A_0_ is the initial cross-sectional area of the hydrogel (mm^2^).

The compressive strain (ε_c_) was estimated as follows:

$$\varepsilon_{c}=\frac{h_{0}-h}{h_{0}}$$

where h_0_ is the original height of the hydrogel and h is the compressed height.

After identifying the point of fracture, data were collected on the maximum compressive stress (fracture stress, σ_c,f_) and the maximum compressive strain (fracture strain, ε_c,f_) during the test. The elasticity modulus (E_c_) was determined as the ratio of compressive stress to compressive strain and corresponds to the slope (tan α) between two points on the stress–strain curve. The toughness (also referred to as dissipated energy, U_cU_) was calculated as the area under the stress–strain curve, representing the fracture energy (W_f_) at the breaking point. All tests were performed at room temperature.

### 4.11. Cell Cytotoxicity Assay In Vitro

The MTT assay was performed to evaluate the metabolic activity and viability of MCF-7 breast cancer cells exposed to Tetra PEG-BSA, Glu-BSA, Heat-BSA, and GDL-BSA hydrogels. Cells were incubated with hydrogel extracts at 0.05%, 0.10%, 0.50%, 1%, 5%, 10%, 25%, 50%, and 100% (*v*/*v*) for 24, 48, and 72 h. Following incubation, 20 μL of MTT dye (Invitrogen, Molecular Probes Inc., Eugene, OR, USA, 5 mg/mL in PBS) was added to each well, and plates were incubated in the dark at 37 °C for 4 h in a 5% CO_2_ incubator.

After incubation, the media were aspirated, and 100 μL of dimethyl sulfoxide (DMSO) was introduced to dissolve the formazan crystals formed by viable cells. The color intensity of the wells was measured at 570 nm using a microplate reader (Bio-Rad Laboratories, Hercules, CA, USA) against a blank reagent; 100% DMEM served as the negative control, while a serial dilution of DMSO starting at 100% was used as the positive control.

Cellular viability (%) was calculated using the following equation:

$$\mathrm{Cellular}\;\mathrm{viability}\left(\%\right)=\frac{\mathrm{Absorbance}\;\mathrm{of}\;\mathrm{treated}\;\mathrm{cells}}{\mathrm{Absorbance}\;\mathrm{of}\;\mathrm{negative}\;\mathrm{control}}\times 100\%$$

This method allowed for a quantitative assessment of the cytotoxicity and biocompatibility of the tested hydrogels over time.

### 4.12. Statistical Analysis

Statistical analysis data are presented as mean ± standard deviation. Duncan’s test was used to determine significant differences at a significance level (α) of 0.05. Statistical analysis was performed with Origin 2018 software. All experiments were performed three times.

## Figures and Tables

**Table 1:** The gelation time and transmittance of different methods.

	Tetra-PEG-BSA Gel	GLU-BSA Gel	HT-BSA Gel	GDL-BSA Gel
Time	11 ± 1 ^a^ (s)	14,400 ± 60 ^b^ (s)	17 ± 2 ^c^ (s)	21,600 ± 78 ^d^ (s)
Transmittance (600 nm)	92.92 ± 0.008 ^a^	72.48 ± 0.598 ^a^	4.80 ± 0.000 ^c^	3.13 ± 0.001 ^c^

Superscript letters a–d indicate statistically significant differences among the samples within the same row (*p* < 0.05). Values in the same row sharing the same letter are not significantly different, whereas values with different letters differ significantly.

**Table 2:** Maximum gel strength (N) of four gelling methods in four swelling solutions.

	Tetra PEG-CHO	Glutaral	Heat-Treated	GDL
Water	3.16 ± 0.66	3.33 ± 0.23	2.78 ± 1.08	1.05 ± 0.05
Gnd HCl	3.43 ± 0.20	4.60 ± 0.05	2.63 ± 0.51	0.63 ± 0.09
Urea	3.26 ± 0.26	2.32 ± 0.27	0.92 ± 0.18	no gel after swelling
SDS	2.03 ± 0.04	1.03 ± 0.16	1.14 ± 0.02	no gel after swelling

**Table 3:** Comparison of compressive properties of four hydrogels.

Gel Samples	ε_c,f_ (%)	σ_c,f_ (kPa)	E_c_ (Pa)	W_f_ (kJ·m^−3^)
Tetra PEG-BSA	84.12 ± 0.76	28.64 ± 1.21	14.7 ± 2.4	468.0 ± 34.9
GLU-BSA	52.70 ± 0.58	9.57 ± 0.32	44.0 ± 4.5	182.8 ± 17.2
HT-BSA	69.92 ± 1.17	17.53 ± 0.54	30.7 ± 2.1	357.9 ± 10.3
GDL-BSA	45.39 ± 0.54	2.23 ± 0.30	34.6 ± 3.7	50.3 ± 2.6

## Data Availability

The original contributions presented in this study are included in the article. Further inquiries can be directed to the corresponding author(s).
